# The news media and the agenda for noncommunicable diseases before and during the COVID-19 pandemic: Losing the competition for coverage and framing responsibility for action in Malawi

**DOI:** 10.1371/journal.pone.0341285

**Published:** 2026-07-24

**Authors:** Dereck L. Hamunakwadi, Stephanie L. Smith

**Affiliations:** School of Public and International Affairs, Virginia Tech, Blacksburg, Virginia, United States of America; Freelance Consultant, Myanmar, MYANMAR

## Abstract

Chronic noncommunicable diseases (NCDs, such as cardiovascular and diabetes) pose an increasing burden in low-and middle-income countries, accounting for ~80% of premature (under age 70) NCD deaths globally. Despite their significant and rapidly growing burden—and status under United Nations Sustainable Development Goal 3.4—little is known about the agenda status of NCDs in lower-income countries. Though coverage of health issues shapes policymaker and public awareness and opinions, news media is an under-analyzed agenda setting arena. This study explores levels of priority for 10 NCDs compared to four infectious diseases and their burdens in the news media arena in Malawi; shifts in the agenda during the COVID-19 emergency; and media framing of actors responsible for addressing Malawi’s NCD burden. The study is informed by analysis of the contents of headlines (an indicator of media priority) and a purposefully selected subset of articles (framing analysis) published in two news outlets between 2015 and 2023. News media priority for NCDs in Malawi remained relatively low despite elevation to Sustainable Development Goal status. Between 2015 and 2019, news media priority in Malawi heavily favored HIV/AIDS, tuberculosis and malaria over 10 NCDs. Media priority shifted to COVID-19 and headlines on mental health surged ahead of those on HIV/AIDS and tuberculosis during the emergency (2020–2023). Alignment between the burden of disease and media priority was inconsistent. News media framed nongovernmental actors (primarily domestic) as responsible for addressing NCDs (predominantly cancers) far more frequently than other actors, including government, politicians and development partners. Featuring patterns of low attention and framing that fails to hold authorities responsible for addressing NCDs, media priority appears unlikely to substantially shift public opinion or policymaker agendas in their favor in Malawi. This study sets the stage for further analysis of media priority for health issues in lower-income countries.

## Introduction

Eighty percent of premature mortality (under age 70) from noncommunicable diseases (NCDs, such as cardiovascular, diabetes, cancers, and mental health disorders) occurs in lower income countries [1]. In the WHO African Region, NCDs are emerging as a leading cause of death, accounting for 37% of mortality in 2019 [2]. NCDs are expected to surpass communicable, maternal, neonatal, and nutritional diseases as the leading cause of death in Sub-Saharan Africa by 2030 [3]. Despite their significant and rapidly growing burden—and status under United Nations Sustainable Development Goal (SDG) 3.4 to reduce it—little is known about the agenda status of NCDs in lower-income countries. News media forms an important and under-analyzed agenda setting arena in which diseases compete for coverage that shapes policymaker and public awareness and opinions [4–8]. This study offers novel insights to priority for NCDs relative to high-profile infectious diseases—and which types of actors are portrayed as responsible for alleviating the NCD burden—in a subset of the news media arena in Malawi.

The exploratory study is guided by three research questions. First, how does the level of agenda status (or priority) for NCDs in the news media arena in Malawi compare to that of high-profile infectious diseases before and during the COVID-19 pandemic? Second, does priority align with the burden of each disease? Third, to learn what kinds of expectations news media coverage may set for addressing NCDs, which actors are framed as responsible for alleviating their burden? The study analyzes priority for 10 NCDs, including a set advanced by the Government of Malawi in alignment with the World Health Organization (WHO) NCD 5x5 and five other priority conditions identified by the Malawi NCDI Poverty Commission [9] in comparison to the three Global Fund diseases (HIV/AIDS, tuberculosis and malaria) and COVID-19 via analysis of coverage in two news media outlets in Malawi.

The news media is one of several interacting arenas for global health agenda setting. In an arenas model, health issues compete for status that is defined by varying levels of resource allocations, such as headlines in the news media arena [5–8]. Well-funded infectious diseases like HIV/AIDS, tuberculosis, and malaria tend to attract a good deal of media coverage, reinforcing their already high profiles [4]. In global health, priority is sometimes aligned with international development norms, such as those advanced by the Millennium and Sustainable Development Goals [6,10]. A rational model suggests priority should be aligned with the burden of disease [8]. Focusing events such as natural disasters and disease outbreaks sometimes rapidly shift agendas and are likely to have done so in the case of the COVID-19 pandemic [5,11]. These propositions from scholarship are under-investigated in media arenas in lower-income countries.

Agenda status in the news media arena is important because health policy decisions are influenced by socio-political contexts—news media play roles in informing policymakers and shaping discussions on public health [4,12,13]. News outlets translate complex health and science information into accessible and easily understood forms [14]. News media portray issues in ways that influence how the public develops schemas and priorities [15]. The arena also helps set expectations for who is responsible for addressing problems. This occurs through framing—portraying problems in ways that assign causal attribution, blame, and responsibility for their redress [16,17]. Such framing may increase the likelihood of issues rising on agendas.

## Methods

This exploratory study analyzed (1) the level of priority for a set of NCDs compared to Global Fund diseases and COVID-19 in a subset of the news media arena in Malawi; (2) alignment with the disease burden and Development Assistance for Health; and (3) how news media frames responsibility for addressing them. We chose a study period (2015−2023) that spans the Sustainable Development Goals and COVID-19 eras. Following guidance from the Global Health Agendas Project, we used headlines to measure disease priority levels in Malawi’s news media arena [5,7,18,19]. We systematically searched the Access World News database for headlines on included diseases. The database contained articles from three news media outlets in Malawi—we included only the two outlets for which data covered the entire study period (*Maravi Post* and *Nyasa Times*).

Diseases and headline search terms are documented in Table 1. We included a set of infectious diseases alongside NCDs because agenda status is best assessed comparatively. We included: (1) the high-profile Global Fund diseases (HIV/AIDS, tuberculosis and malaria) to assist with gauging the relative degree of news media priority for NCDs; (2) COVID-19 as a focusing event that was likely to have reordered disease priorities in the arena [5] and (3) NCDs that WHO (under the NCD 4x4 and 5x5 rubrics) and the Malawi NCDI Poverty Commission [9] have worked to advance as priorities in Malawi.

**Table 1:** Diseases and search terms.

WHO 5x5 NCDs	Cancer	Cardiovasc. disease	Chronic respiratory diseases (CRDs)	Diabetes	Mental Health
*Search Terms*	Cancer* OR neoplasm*	Cardiovascular OR hypertension OR “blood pressure” OR “heart attack” OR stroke	“Chronic obstructive pulmonary disease*” OR COPD* OR Asthma* OR “occupational lung disease*” OR pulmonary hypertension	Diabetes OR diabetic	“mental health” OR depression OR suicide OR anxiety OR “substance use” OR “substance abuse” OR “alcohol abuse” OR addiction
Malawi NCDI Poverty Commission	**Sickle cell**	**Ulcers**	**Digestive disorders**	**Neurological disorders**	**Musculoskeletal disorders**
*Search Terms*	Sickle cell	Ulcer*	Digestive disorder*	Neurologic* disorder* OR Epilepsy* OR dementia* OR Alzheimer*	Musculoskeletal disorder*
Global Fund Diseases and COVID-19	**Tuberculosis**	**HIV/AIDS**	**Malaria**	**COVID-19**	
*Search Terms*	Tuberculosis* OR TB*	HIV* OR AIDS*	Malaria*	COVID-19* OR Coronavirus*	

For transparency and to facilitate replication, data were collected using systematic searches in the Access World News by NewsBank database [20] as follows: headline (disease terms in Table 1); source location (Malawi); source name (Maravi Post; Nyasa Times); date selector (by year for 2015−2023). In addition to well-known and commonly used terms for the Global Fund diseases and COVID-19, we used search terms identified in the Malawi NCDI Poverty Commission [9] report. We tried searching for some specific medical conditions, such as coronary and cerebrovascular disease, with no results. We had better results with terms in common/lay use, such as dementia and Alzheimer for neurological disorders, heart attack for cardiovascular disease, and addiction for mental/behavioral health. We used these to expand search coverage. Our findings are limited by the search terms used (which were not exhaustive) and by our focus on headlines. Our use of headlines was strategic, however. Headlines form a more robust indicator of priority than broader article contents, which may only mention an issue and not focus on it.

For the analysis of how news media frames actor responsibility for addressing NCDs in Malawi, we included only articles that focused on NCDs in the Malawi context (Table 2). For example, articles on World Diabetes Day observances in Malawi were included while those on American celebrities with cancer diagnoses were excluded. We analyzed the contents of articles meeting the inclusion criteria for reporting on actors portrayed as addressing one or more NCDs in Malawi by committing stated (verbal or written), institutional (plans, programs, staff) and/or budgetary resources following the Political Commitments Framework [21,22]. We used the framework to create a strong measure of responsible actor frames, a methodological innovation.

**Table 2:** Total headlines and articles featuring responsibility frames by disease.

	Diseases and disorders	Total headlines (n=)	Headlines focus on NCDs in Malawi (n=)	Frames responsibility for addressing NCDs in Malawi (n=)
**WHO 5x5**	*Cancers*	124	81	71
	*Cardiovascular*	24	12	7
	*Diabetes*	20	16	15
	*Respiratory*	0	0	0
	*Mental health*	219	126	47
**Malawi NCDI Poverty Commission**	*Digestive*	0	0	0
	*Muskuloskeletal*	0	0	0
	*Neurological*	6	4	0
	*Sickle cell*	0	0	0
	*Ulcers*	1	1	0
**Global Fund**	*HIV/AIDS*	247	Not applicable	Not applicable
	*Malaria*	73	Not applicable	Not applicable
	*Tuberculosis*	213	Not applicable	Not applicable
**Focusing event**	*COVID-19*	1237	Not applicable	Not applicable

Authors’ analysis of data from Maravi Post and Nyasa Times collected using Access World News by NewsBank [20].

Procedurally, we created an initial codebook that identified types of NCDs (Table 1), health policy actors and responsibility frames (aligning with the Political Commitments Framework). We coded a pilot sample of articles on cancer (n = 25) and mental health (n = 25). We discussed any inconsistencies and refined the codebook accordingly to enhance intercoder reliability. We met regularly to discuss coding and questions about how to code specific articles, updating the codebook as we analyzed articles meeting criteria for inclusion.

We also categorized actors by type to assess which are most and least actively framed as responsible. Types of actors included government, law enforcement, industry, nongovernmental, development partners, politicians, researchers and service providers. Specific examples of actors and the kinds of resources they committed for each issue are reported alongside total numbers in the Findings section.

This is an exploratory study designed to offer insights to under-analyzed dimensions of national-level health agenda setting, and to show how status might be measured via headlines in domestic media arenas. The analysis does not capture other dimensions of media priority, such as sentiment, prominence among a broader set of policy issues, or wider media circulation (including social media, broadcast media, and other print and online news media). Our ability to systematically collect and analyze data from major news outlets in Malawi was limited by its accessibility and format. For some of the major news outlets, we could search for the diseases but could not assess completeness of results (e.g., years available) or systematically organize and analyze them. We used the *Maravi Post* and *Nyasa Times* (online news sources) because we could systematically collect and analyze data from them by using Access World News, which archives news content from more than 200 countries and territories, potentially facilitating future comparative research.

We did not delve into the complete media ecosystem in Malawi for this exploratory study (see Jimmy Kainja’s chapter in *The Palgrave Handbook of Democracy, Governance and Justice in Africa* for an historical account [23]). The contents of the two news outlets included in this study are not representative of coverage in the wider media landscape. However, we checked the consistency of content published in *Maravi Post* [24] (circulation unreported) and *Nyasa Times* [25] (8 million hits per month reported) against a random sample from *The Nation/Nation Online* [26]—the major press that offered the best access to content—between 2017 (the earliest year available) and 2023. The order of priority for the six diseases with the most headlines was closely aligned, with COVID-19 the leader (n = 1,237 headlines in *Maravi Post* and *Nyasa Times* combined and n = 883 in *Nation Online* [26]). We calculated ratios to further check comparability of coverage. The ratios of headlines on COVID-19 to the next five diseases were roughly comparable: COVID-19 to HIV/AIDS 5:1 in *Maravi Post* and *Nyasa Times* combined/4.9:1 in *Nation Online*; mental health 5.6/5.2; tuberculosis 5.8:1/4.3:1; cancers 10/10.2:1; malaria 17/14:1. The order and figures diverge for headlines on the other eight NCDs, which is to be expected with much lower coverage (*Maravi Post* and *Nyasa Times* combined headlines n = 47; range = 0–24; mean = 5.9; median = 0.5) (*Nation Online* headlines n = 78; range = 0–24; mean = 9.75; median = 0). In sum, the data used in this exploratory study do not meet criteria for representativeness, but they form a reasonable basis for offering preliminary insights to media priority for the set of diseases in Malawi and our aims to develop methods for assessing priority levels via analysis of headlines.

## Findings

Informed by analysis of headlines published in two outlets covering the news in Malawi, we first present findings on news media priority for 10 NCDs compared to three Global Fund diseases and COVID-19 just before (2015−2019) and during the health emergency (2020−2023) and aggregated over the full period. We next compare media priority to the burden of disease, finding some consistency (both in the top tier for HIV/AIDS, COVID-19 and cancers) and some misalignment (cardiovascular diseases high burden but lower media priority). The section concludes with findings from analysis of the subset of articles that framed specific actors as responsible for alleviating the NCD burden in Malawi, finding an emphasis on nongovernmental actors (especially domestic civil society) rather than those with substantial authority and resources to address NCDs at scale.

### News media priority for NCDs compared to Global Fund diseases and COVID-19 in Malawi

News media priority for NCDs lagged priority for three Global Fund diseases and COVID-19 in Malawi, as indicated by total headlines in *Maravi Post* and *Nyasa Times* between 2015 and 2023 (Table 2). During the pre-COVID-19 era (2015−2019), headlines indicative of media priority for Global Fund diseases (n = 310) outnumbered those on all 10 NCDs included in this study (n = 197) by a 1.6:1 ratio. During the COVID-19 era (2020−2023), headlines on COVID-19 (n = 1,237) exceeded those on Global Fund diseases (n = 223) by a 5.5:1 ratio and NCDs (n = 197) by a 6.3:1 ratio. During the pandemic period, the ratio of Global Fund diseases to NCDs shrank to 1.1:1.

**Fig 1:**
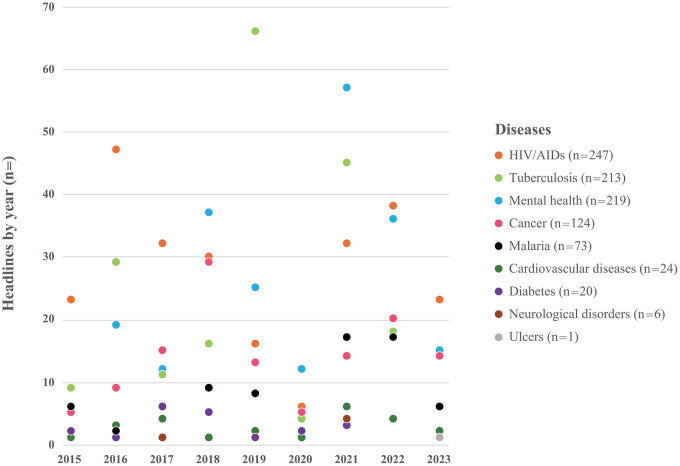
Media headlines on NCDs and Global Fund diseases in Malawi, 2015-2023. NCDs with n = 0 headlines include chronic respiratory diseases, musculoskeletal disorders, digestive disorders and sickle cell. Authors’ analysis of data from Maravi Post and Nyasa Times using the Access World News by NewsBank database [20].

**Fig 2:**
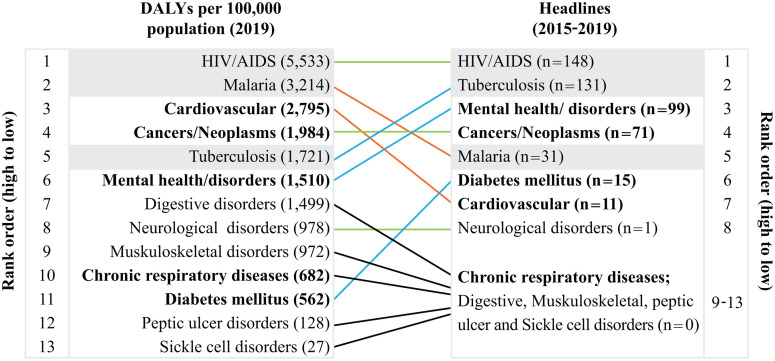
Media priority and burden of disease in Malawi during the immediate pre-COVID-19 period. Infectious diseases are highlighted in gray, NCD 5x5 are bolded and other NCDs appear in plain text. Color-coded lines are used to show for which issues media priority is higher (blue), lower (orange), at the same level (green) or nonexistent (black, n = 0) compared to the burden of disease. Authors analysis of headlines in *Maravi Post* and *Nyasa Times* from Access World News [20]; IHME [27] Global Health Data Exchange.

**Fig 3:**
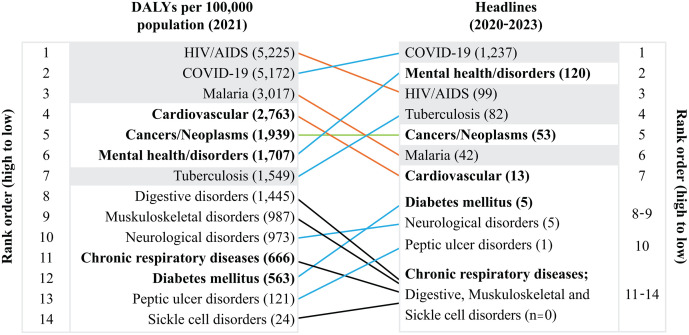
Media priority and burden of disease in Malawi during the COVID-19 era. Infectious diseases are highlighted in gray, NCD 5x5 are bolded and other NCDs appear in plain text. Color-coded lines are used to show for which issues media priority is higher (blue), lower (orange), at the same level (green) or nonexistent (black, n = 0) compared to the burden of disease. Authors analysis of headlines in *Maravi Post* and *Nyasa Times* from Access World News [20]; IHME [27] Global Health Data Exchange.

Fig 1 shows the number of headlines for each disease by year except for COVID-19, which was a media priority outlier on a different scale. Total headlines by disease for the pre- and during COVID-19 periods are shown in Figs 2 and 3. Between 2015 and 2019, headlines indicative of media priority for HIV/AIDS outpaced those on tuberculosis (1.1:1 ratio), followed by mental health (1.5:1 ratio), cancers (2.1:1 ratio), malaria (4.8:1 ratio), diabetes (9.9:1 ratio) and cardiovascular diseases (13.5:1 ratio). There were one or no headline results for the other NCDs, suggesting a particularly low or nonexistent level of media priority in the two outlets included in this study.

Between 2020 and 2023, headlines on COVID-19 (n = 1,237) outpaced those on second place mental health (10:1 ratio), HIV/AIDS (12:1 ratio), tuberculosis (15:1 ratio), cancers (23:1 ratio), malaria (29:1 ratio) and cardiovascular diseases (36:1 ratio) (Fig 3). The order of priority shifted at the disease level between periods, with mental health moving ahead of HIV/AIDS and tuberculosis. Few articles (and in some cases none) were published on other NCDs, suggesting very low levels of media priority for them. Overall, priority as indicated by headlines increased for mental health disorders, neurological disorders and malaria while it declined or remained dormant for most others in the two outlets included in this study.

### News media priority compared to the burden of disease in Malawi

Figs 2 and 3 compare the order of media priority to the burden of disease using DALYs (Disability-Adjusted Life Years) as a measure. Though the set of diseases and disorders is limited, the Figures begin to reveal how media priority diverged from the disease burden during both periods. Before the emergence of COVID-19, all three Global Fund diseases and cancers were in the top five for burden and headlines. Cardiovascular diseases were ranked third highest for disease burden (and highest burden among NCDs) but lower for media priority (seventh) with only n = 11 headlines between 2015 and 2019. Mental health and digestive disorders posed nearly the same disease burden (~1,500 DALYs, ranking seventh and eighth respectively on the list)—mental health ranked higher (third) for headlines while digestive disorders were unranked with no headlines. Only cancers (fourth) and neurological disorders (eighth) were equivalently ranked.

Between 2020 and 2023, COVID-19 soared to the top of both lists—posing a high burden and becoming highly prioritized in the news media arena. The burden of disease changed little otherwise except for mental health disorders, which increased in DALYs and moved ahead of HIV/AIDS and tuberculosis in media priority. The COVID-19 focusing event may have displaced some news media priority for the Global Fund diseases while increasing priority for mental health may be partly explained by its impacts. Twelve articles directly linked mental health with COVID-19.

#### News media priority compared to Development Assistance for Health in Malawi.

**Fig 4:**
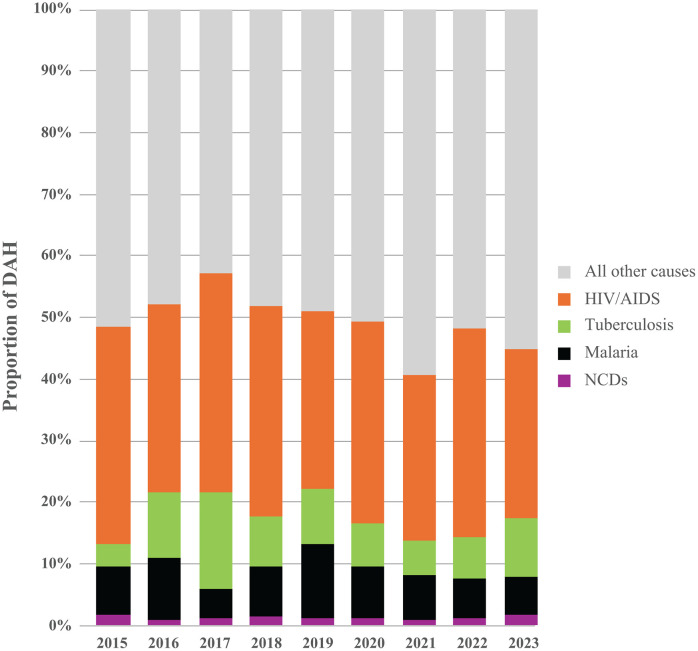
Proportion of Development Assistance for Health to NCDs, HIV/AIDS, tuberculosis and malaria, 2015-2023 [27].

Lower overall news media priority for NCDs compared to the Global Fund diseases and COVID-19 generally aligns with the distribution of Development Assistance for Health (DAH) in Malawi. On average, approximately half of all DAH went to the Global Fund Diseases between 2015 and 2023 while NCDs received less than 2% [27]—a 25:1 ratio that is far higher than the 1.6:1 news headline ratio. DAH to HIV/AIDS (mean 31.4%, range 26.9–35.4%), tuberculosis (mean 8.0%, range 3.7–15.9%) and malaria (mean 8%, range 4.6–12.1%) on an individual basis also far outpaced that to NCDs as a group (mean 1.3%, range 1.0–1.6%) (Fig 4) [27]. DAH is not broken down for individual NCDs.

### Framing responsibility for addressing NCDs in Malawi in the national news media arena

Portrayals of responsibility for alleviating the burden of health problems contributes to shaping public and policymaker expectations for action. We analyzed the subset of articles featuring headlines on NCDs in Malawi for portrayal of actors responsible for addressing them. Of the n = 238 articles that featured headlines on NCDs in Malawi between 2015 and 2023 in the *Maravi Post* and *Nyasa Times*, approximately 60% (n = 140) portrayed specific actors as responsible for addressing NCDs included in this study by making stated, institutional or budgetary commitments (Table 2). About half of the articles featuring these strong responsibility frames focused on cancer (n = 71), followed by mental health (n = 47), diabetes (n = 15) and cardiovascular diseases (n = 7). We did not observe assignment of responsibility for the other NCDs.

**Fig 5:**
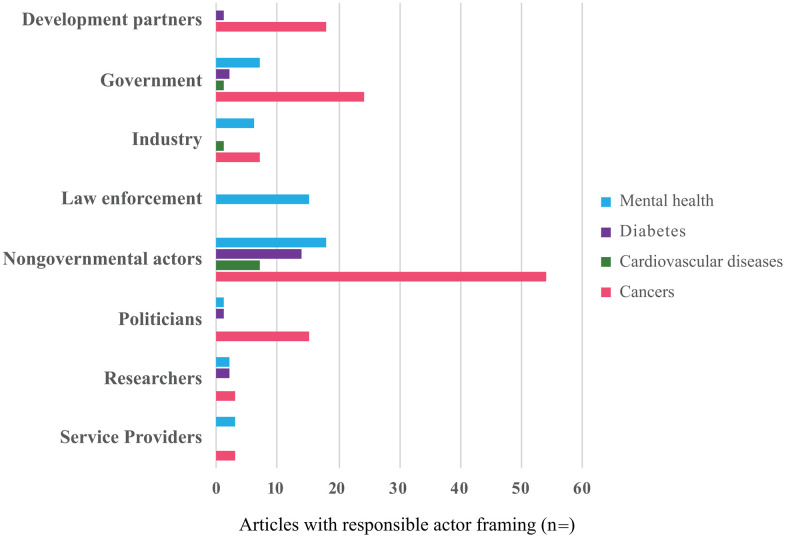
News articles framing responsibility by NCD and category of actor in Malawi, 2015-2023. Authors’ analysis of data from Maravi Post and Nyasa Times using Access World News by NewsBank [20].

**Fig 6:**
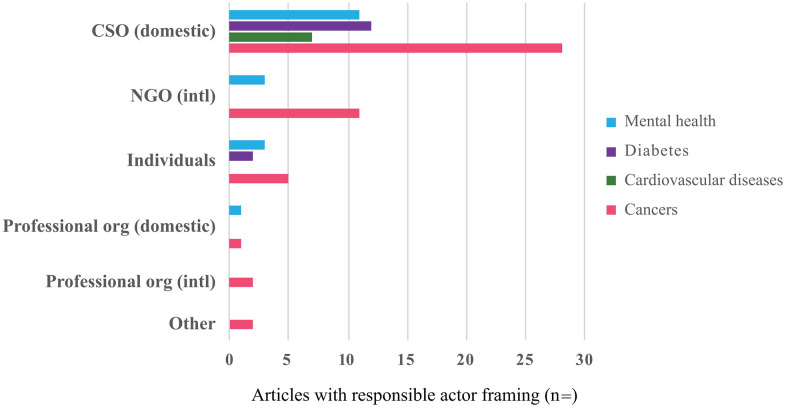
News articles framing responsibility by NCD and category of nongovernmental actor in Malawi, 2015-2023. Authors’ analysis of data from Maravi Post and Nyasa Times using the Access World News by NewsBank database [20].

**Fig 7:**
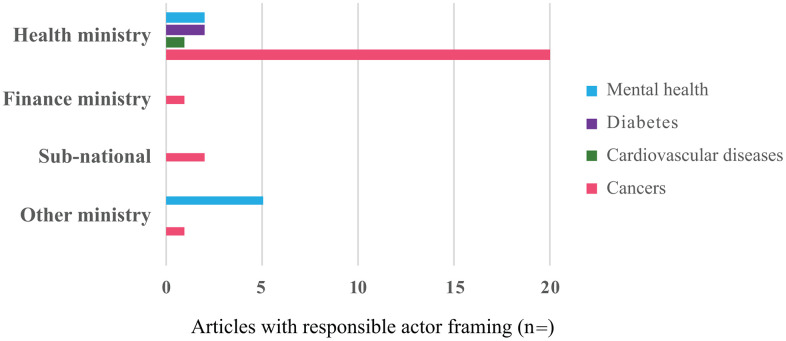
News articles framing responsibility by NCD and category of government actor in Malawi, 2015-2023. Authors’ analysis of data from Maravi Post and Nyasa Times using the Access World News by NewsBank database [20].

Figs 5 through 7 provide high level overviews of the categories of actors framed as responsible for addressing NCDs in Malawi. Fig 5 shows that reporting focused foremost on nongovernmental actors followed by government actors, development partners (primarily for cancer), law enforcement (concentrated in mental health coverage), industry, politicians, researchers and service providers. Figs 6 and 7 show a further breakdown for the two categories of actors most frequently framed as prioritizing NCDs by committing resources: nongovernmental actors (mostly domestic CSOs, international NGOs and private individuals) and government actors (primarily the Ministry of Health and Population). Findings on responsible actor frames are organized by disease in the order of most to fewest articles along with several examples in the subsections that follow.

### Framing responsibility for cancer in Malawi’s news media arena (n=71 articles)

Responsibility and commitment framing for cancers focuses heavily on nongovernmental actors (Figs 5 and 6), including domestic CSOs (n = 28 articles, with Think Pink Malawi n = 11 and WOCACA n = 6 identified in more than half of articles); international NGOs (n = 11, such as Malawi Health Care Support and Empowerment Cancer Advocacy Network (n = 2 each); several individuals (n = 10); and a few professional associations (n = 1 domestic, n = 2 international). Much of the reporting focuses on cervical cancer awareness and screening including, campaigns carried out by the Hope for Cancer Foundation, Empowerment of Women and Girls, WOCACA (Women’s Coalition Against Cancer) and Organization of African First Ladies [28,29]. Former Miss Malawi Blandina Khondowe’s Think Pink Campaign’s investments in improving breast and cervical cancer awareness and treatment attracted more media attention than any other nongovernmental actor [30,31].

Government actors are also frequently portrayed as responsible—the Ministry of Health and Population leads in coverage (n = 20 articles) with infrequent reporting on responsibility and commitments by other government actors (n = 1 each on the Finance and Homeland Security ministries, and n = 2 on sub-national governments) (Figs 5 and 7). Representatives of the Ministry of Health and Population are cited for making commitments to two major initiatives. One is to address cervical cancer, expressing desire to reach girls with HPV vaccination, providing the vaccine, and regularly screening women in district hospitals, for example [32–35]. Another is to develop a national cancer center [36,37].

Commitments from international development partners are far more frequently reported on for cancers than any other NCD included in the study (n = 18 of 19 articles identifying partners). Most development partners, including GAVI, the Global Fund, UNFPA, UNICEF, USAID and WHO, are only mentioned in one or two articles. For instance, one article reported that the Global Fund and UNICEF funded Malawi’s cervical cancer vaccine program [32].

Commitments by politicians (n = 15 articles) are reported more frequently to address cancers than other NCDs, with former First Lady and Malawi Health Ambassador Gertrude Mutharika leading the pack. For instance, the former First Lady mainly contributed to fighting cancer in the country through awareness campaigns involving cervical and breast cancer screening [38,39]. Other politicians include the family of former First Lady Annie Muluzi (who died of cancer in 2021), which announced plans to construct a cancer center in her honor [40]. President Chakwera also appealed for financial resources to construct cancer treatment facilities in Malawi [41].

In the private for-profit sector (n = 7 articles), several donations and campaigns were reported. For instance, Amgen Pharmaceutical Company donated chemotherapy and other medications [42], the National Bank of Malawi donated funds to support cancer screening and treatment [43,44], and Airtel (a telecommunications company) donated cervical cancer screening materials [45]. Few articles reported on cancer researchers (n = 3) or service providers (n = 3).

### Framing responsibility for mental health in Malawi’s news media arena (n=47 articles)

Like for cancer, coverage framing responsibility for mental health in the *Maravi Post* and *Nyasa* *Times* mainly focuses on non-governmental actors (Figs 5 and 6). The actors include domestic CSOs (n = 11 articles, including Connect Plus Resource Institute n = 4, Lilongwe Music and Arts Festival n = 3, and Caring Hands Center Limited n = 2); international NGOs (n = 3 articles, such as Mental Care UK n = 2 and Lifeline International); and individual mental health advocates including, a psychologist, a United Kingdom-based mental health specialist, and an Afro-spiritual musician. Much of the reporting includes campaigns and innovative initiatives to raise awareness of mental health problems in Malawi. This consists of the campaign for the decriminalization of suicide and increasing funding for mental health services by the Connect Plus Resource Institute’s Chief Executive Officer [46] and the country Deputy Director of Lifeline African Network for Protection and Prevention of Child Abuse and Neglect-Malawi [47]; the use of music and entertainment to address mental health and gender-based violence by the Lilongwe Music and Arts Festival [48]; and the partnership between Caring Hands Center Limited and SPARC Systems Limited information technology company to develop a mental health awareness app [49].

Law enforcement actors, especially the Malawi Police Service, are also frequently portrayed as responsible for addressing mental health problems in Malawi (n = 15 articles) (Fig 5). On the one hand, the two outlets portray police as criminalizing suicide in line with Section 229 of the Malawi Penal Code. For instance, a Chikwawa Police Station Public Relations Officer was quoted as saying that attempted suicide carries a maximum sentence of seven years imprisonment [50]. On the other hand, law enforcement is portrayed as encouraging people with mental health problems to seek help through counseling [51,52] to prevent suicide.

Government actors were less frequently portrayed by the media outlets as responsible for addressing mental health issues (n = 5 articles) (Figs 5 and 7). The major government actor portrayed as mobilizing resources to address mental health issues is the Ministry of Health (n = 2 articles). Other government actors include the Central East Police Region Women Network, the Malawi Human Rights Commission, the Ministry of Gender, Children, Community Development and Social Welfare, the Malawi Council for the Handicapped, and the Director of Youth in the Ministry of Youth, Sports, Labor and Manpower Development. Government officials are identified as making stated, institutional and budgetary commitments during anti-suicide campaigns [53,54], World Mental Health Commemoration Day (n = 6 articles) [55], and the introduction of mental health forums to address suicide [54].

Various private for-profit sector actors were reported to address mental health problems (n = 6 articles) (Fig 5). They include the National Bank of Malawi (n = 2), Ecobank Malawi, Sweat Factory Fitness Studio, Macadamia Entertainment and SPARC Systems Limited ICT company. The actors are reported to have raised mental health awareness in various ways, including introducing mental health booths in universities [56], hosting fitness events to raise funds [57] and organizing a Youth Movement for Mental Health Awareness [58].

Researchers (n = 2) and Service providers (n = 3) were less frequently portrayed in the as prioritizing the mental health problem in the outlets (Fig 5). Notably, St. John of God Hospitaller Services (n = 3 articles) pledged institutional and budgetary resources to support mental health by housing 120 people experiencing suicidal thoughts per month [59], increasing and training mental health services personnel [60], and conducting community outreach programs to raise awareness on suicide [61]. Among researchers, St. John of God College of Health Sciences is portrayed as raising mental health awareness on behalf of people living in suburban areas [62].

Politicians were rarely portrayed by the media outlets (n = 1 article) as committing resources to improve mental health (Fig 5). There was one exception. The Member of Parliament for Lilongwe South, Peter Dimba, tried to convince other parliamentarians to decriminalize attempted suicide [47].

### Framing responsibility for diabetes in Malawi’s news media arena (n=15 articles)

Coverage framing responsibility for addressing diabetes (n = 15 articles) by the two news outlets also primarily focus on nongovernmental actors (Figs 5 and 6). They include domestic CSOs (n = 12 articles) and individual advocates (n = 2 articles). The domestic CSOs include Community Against Diabetes & Hypertension (n = 6), Diabetes Association of Malawi (n = 2), Journalist Association against AIDS (Journalaids) (n = 2), and Bwaila Lions Club (n = 1). For example, the Bwaila Lions Club conducted diabetes awareness campaigns encouraging screening [63]. The Diabetes Association of Malawi hosted concerts to raise diabetes awareness and raised funds to buy equipment for Queen Elizabeth Central Hospital [64]. The Community against Diabetes & Hypertension campaigned for diabetes awareness [65], raised funds to open a counseling center for people living with NCDs [66], and opened diabetes clubs across the country [67]. And Journalaids pushed to integrate a diabetes curriculum in primary schools [68].

Government actors (n = 2) and researchers (n = 2) were less frequently portrayed as responsible for addressing diabetes in Malawi. Government actors included the NCD and Mental Health Unit and the broader Ministry of Health. They conducted a press conference on World Diabetes Day and hosted stakeholder meetings [64].

The media outlets rarely portrayed politicians (n = 1) or international development partners (n = 1) as responsible for prioritizing diabetes. Then Chairperson of the Parliamentary Committee on Health Juliana Lunguzi was reported to have promised Malawians that her committee would lobby for more funding for NCDs such as diabetes [67]. And the World Diabetes Foundation of Denmark was reported to fund JournAIDS to implement a diabetes prevention project [68].

### Framing responsibility for cardiovascular diseases in Malawi’s news media arena (n=7 articles)

Articles framing responsibility and commitments for cardiovascular diseases primarily focus on domestic CSOs under the nongovernmental actors category (n = 7 articles), followed by government (n = 1) and private for-profit actors (n = 1) (Fig 5). Community Against Diabetes and Hypertension (n = 5) and Stroke Support Organization (SSO) (n = 2) appeared most frequently. The coverage mainly focused on awareness and screening campaigns, including a partnership between the Stroke Support Organization (a CSO) and Malawi Stroke Unit (government) [69]. Community Against Diabetes and Hypertension developed diabetes and hypertension peer support groups in Lilongwe [70] and opened a center for counseling, examination and testing for hypertension and diabetes [67]. First Capital Bank’s (private for-profit sector) head of marketing reported a donation of MK3.5 million (Malawian Kwachas) to help support the Stroke Support Organization’s work [71].

## Discussion

Policymaker and public expectations for action on health issues may be affected by news media coverage of them [4,12,13]. This study explores levels of media arena priority for 14 high-burden diseases and disorders in Malawi in relationship to a set of propositions from scholarship concerning likely influences on their status, including disease burden, funding levels, and focusing events. It also analyzes and offers new insights to ways in which actor responsibility for addressing the outsized burden of NCDs in Malawi is framed in news media outlets. To our knowledge, this is the first such analysis of comparative media priority for NCDs in a lower-income country setting. The findings form a mix of the expected and surprising.

Overall, the analysis of headlines indicative of media priority in two news outlets shows that the agenda status of three Global Fund diseases and COVID-19 far exceeded that for 10 NCDs between 2015 and 2023. This was despite the latter’s high and growing burden, SDG 3.4 status and inclusion among Malawi NCDI Poverty Commission priorities. Media priority diverged from funding trends, in which NCDs historically received <2% of DAH to Malawi. Total headlines on mental health and cancers ranked in the top five—ahead of HIV/AIDS, tuberculosis and malaria but behind COVID-19 during the emergency period.

The study provides some evidence that the COVID-19 focusing event precipitated an increase in mental health priority that pushed coverage ahead of HIV/AIDS and tuberculosis in Malawi. During the COVID-19 emergency (2020–2023), media priority as indicated by annual rates of coverage increased for the singular coronavirus, mental health, malaria, cardiovascular diseases and neurological disorders. It declined for tuberculosis, HIV/AIDS, cancers and diabetes mellitus. Media priority for chronic respiratory diseases and for digestive, musculoskeletal and sickle cell disorders remained unregistered in the two news outlets with n = 0 headlines—a finding that likely reflects the issues’ low profiles and methodological limitations of this exploratory study (discussed in the Methods section).

Though preliminary, this study suggests that those with the authority and resources to address Malawi’s NCD problem at scale (politicians, government, development partners) are relatively infrequently portrayed as responsible for action in news coverage. Less-resourced nongovernmental actors—predominantly domestic CSOs—are most frequently portrayed as committing resources to address NCDs in Malawi. If government authorities are not framed as responsible for addressing health problems like NCDs, other research suggests media coverage is unlikely to function as a mechanism for shifting public opinion and policymaker agendas [17].

Most framing of responsibility for addressing NCDs in the two news outlets included in this study focuses on cancers. The media’s framing of mental health as a law enforcement issue is also notable. These emphases in news coverage may serve to advance patterns of priority that favor cancers over other high-burden NCDs and mental health as a criminal over public health issue, respectively, in other agenda setting arenas.

This is an exploratory study based on analysis of headlines and articles published in two news media outlets covering Malawi between 2015 and 2023. The selection of outlets for inclusion was largely driven by the availability of data that could be systematically collected and analyzed—these data aligned with trends found in a random sample from a major press (*The Nation/Nation Online*). The findings are not generalizable. The analysis did not capture the sentiment of coverage or its prominence among headlines on other policy issues. We did not investigate reasons for the patterns observed but suspect they are influenced by several factors, including but not limited to generally low *political* priority for NCDs, sources and volumes of press releases and related events, and a media industry seeking the more spectacular to drive readership and advertising revenue. Drivers of media coverage in lower-income countries should be investigated in future research.

## Conclusion

This study explored underexamined levels and a set of propositions from scholarship regarding priority for 10 NCDs compared to four infectious diseases in a subset of the news media arena in Malawi, contributing preliminary insights to the phenomena in a high-burden lower-income country context and a roadmap for future research. Higher media priority for COVID-19 and the Global Fund diseases compared to most NCDs generally aligns with expectations from scholarship related to focusing events [5,11], funding trends [4], and the burden of disease historically and during an international emergency [5,8]. The study reveals some variation at the disease level, however, with mental health and cancers among the top five health priorities in the news media arena (as represented by headlines studied in two outlets) during periods just before (2015−2019) and during the COVID-19 emergency (2020−2023). There is some evidence that COVID-19 may have crowded in news media priority for mental health—in addition to potential policy impacts, whether and how this may have happened should be further investigated, as should drivers and impacts of news media priority for other health issues.

Combined with relatively low news media priority, the few stories framing those with the authority and resources to alleviate the NCD burden seem unlikely to substantially shift policymaker or public awareness and opinions in their favor. Scholarship suggests that equation might change with a powerful focusing event or advocacy coalition that executes a savvy media strategy considering the interests of publishers and reporters [5–7,11]. Or it might change with increasing priority for NCDs in the political (policy and funding), civil society (public demand), private industry (employer demand), research (new data on cost-effective local solutions) or international aid (funding) arenas [5–8]. Such stimuli and interaction effects are important matters for future research.

Scholars and proponents should examine whether what the Malawi case reveals about low media priority levels and suboptimal responsibility frames for NCDs applies in other low-income country contexts. For scholars, doing so may help to inform analyses of agenda setting dynamics and outcomes as called for by contributors to the Global Health Agendas Project [5–7]. For proponents, doing so may help them address common challenges health networks face, including building strong coalitions and positioning issues in ways that prompt policymakers to act [72].
